# Characterization of recombinant laccase from *Trametes versicolor* synthesized by *Arxula adeninivorans* and its application in the degradation of pharmaceuticals

**DOI:** 10.1186/s13568-019-0832-3

**Published:** 2019-07-11

**Authors:** Katarzyna Litwińska, Felix Bischoff, Falko Matthes, Rüdiger Bode, Twan Rutten, Gotthard Kunze

**Affiliations:** 1 Leibniz Institute of Plant Genetics and Crop Plant Research (IPK), Corrensstr. 3, 06466 Gatersleben, Germany; 2 Jäckering Mühlen- und Nährmittelwerke GmbH, Vorsterhauser Weg 46, 59007 Hamm, Germany; 3 Institute of Microbiology, University of Greifswald, Jahnstr. 15, 17487 Greifswald, Germany

**Keywords:** *Arxula adeninivorans*, Laccase, Degradation, Pharmaceuticals, Yeast

## Abstract

Recent years have seen an increasing interest in laccase enzymes. Due to their ability of oxidizing various substrates, they are nowadays applied in multiple industrial fields including pulp delignification, textile dye bleaching, and bioremediation. In contrast to laccase production from native sources, with its generally low yield and high cost, heterologous laccase expression is far better suited to meet the growing industrial demands. *TVLCC5* gene encoding *Trametes versicolor* laccase 5 was overexpressed in *Arxula adeninivorans* using the strong constitutive *TEF1* promoter. Recombinant Tvlcc5 protein was purified by immobilized-metal ion affinity chromatography and biochemically characterized using 2,2′-azino-bis(3-ethylbenzothiazoline-6-sulphonic acid) (ABTS) as substrate for standard activity assays. The enzyme showed the highest activity at 50 °C between pH 4.5–5.5. The half-life of Tvlcc5 at 60 °C was around 20 min. The negative effect of chloride anions on enzyme activity was demonstrated. A fed-batch cultivation of Tvlcc5 producing strain *A. adeninivorans* G1212/YRC102-TEF1-TVLCC5-6H was performed and resulted in a laccase activity of 4986.3 U L^−1^. To improve the expression level of recombinant laccase in *A. adeninivorans*, cultivation conditions were optimized by single factor experiments. Recombinant Tvlcc5 proved to be a promising agent for degradation of pharmaceuticals that are an important source of environmental pollution. Concentration of diclofenac and sulfamethoxazole decreased to 46.8% and 51.1% respectively after 24 h incubation with Tvlcc5. When 1 mM redox mediator ABTS was added complete degradation was obtained within 1 h.

**Electronic supplementary material:**

The online version of this article (10.1186/s13568-019-0832-3) contains supplementary material, which is available to authorized users.

## Introduction

Annual worldwide intake of active pharmaceutical ingredients (APIs) is estimated at 100,000 tons (Touraud and Roig 2008). Consumption varies strongly between countries but is particularly high in Europe with values between 50 and 150 g per year and capita.

Lipid regulators, antibiotics, antiepileptic and anti-inflammatory drugs as well as β-blockers and antidepressants are amongst the most commonly prescribed pharmaceuticals (Huschek et al. 2004; Fent et al. 2006). Due to the low absorption capacity of most APIs, they leave the human body unaffected or as a metabolite over the renal system, thus ending up in the world’s water system. This problem is intensified through improper disposal of unused or expired medicinal products. It is estimated that approximately 50% of patients in USA and UK store those products in their households and more than half discard them in household waste or flush them down the toilet or sink (Bound and Voulvoulis 2005; Seehusen and Edwards 2006). In EU 50% or more of unused pharmaceuticals is also not returned to the pharmacy (European Environment Agency 2010; BIO Intelligence Service 2013). The amount of unused medicinal products in Germany is assessed to 5700 tons/year. The START survey, published in 2008, showed that nearly half of the German citizens dispose unused liquid medicinal products in drains or toilets, and 15.7% at least occasionally dispose unused or expired tablets in the toilet (Keil 2008). This behavior has led to increasing concentrations of certain APIs in wastewater treatment plants. Natural processes including biological degradation, sorption, air stripping, or phototransformation can remove only part of the APIs and their effectiveness is highly dependent on the environmental conditions of each wastewater treatment plant (Andreozzi et al. 2003; Carballa et al. 2004; Joss et al. 2006).

Processes like ozonation and UV-treatment that have been developed to enhance the degradation processes are under suspicion of causing products with an even higher negative impact on the ecosystem (Okuda et al. 2008; BIO Intelligence Service 2013).

The higher reaction specificity of an enzymatic approach could tackle this problem. In order to eliminate negative effects of pharmaceutically active compounds on the ecological system, one must prevent the binding to their specific receptors. In the present study, laccase was used to achieve this goal. Laccases are multicopper oxidases capable of interacting with a wide spectrum of phenolic compounds, in most cases leading to the formation of radicals. Under low oxygen conditions oxidative polymerization prevails, leading to the formation of polyphenols, which are not recognized anymore by the receptors (Gianfreda et al. 1999; Giardina et al. 2010).

APIs that are not phenolic, but contain benzene-like ring structures, can be transformed into phenolics by cytochrome P450 (CYP450). Expressed in cells of the human liver, CYP450 are naturally involved in the processing of pharmaceuticals in the human body. They increase the solubility of API by hydroxylation, which enables the excretion via the renal system (Wrighton and Stevens 1992; Furge and Guengerich 2006). These hydroxylated pharmaceuticals might be recognized as substrates by the laccase.

The yeast *Arxula (Blastobotrys) adeninivorans* contains several different genes encoding for CYP450 and thus can be employed for the removal of pharmaceutically active compounds in wastewater treatment plants. However, laccase genes are absent from the genome of this yeast. In this study laccase genes from the fungi *Trametes versicolor* and *Pycnoporus cinnabarinus* were expressed in the yeast *A. adeninivorans* with the future aim of generating a whole cell biocatalyst containing the activities of both laccase, attached to the cell surface and CYP450, which are essential for the inactivation of pharmaceutically active compounds from wastewater by oxidative polymerization.

## Materials and methods

### Strains and cultivation conditions

*Escherichia coli* strain XL1-Blue MRF’ Δ(*mcrA*)*183* Δ(*mcrCB*-*hsdSMR*-*mrr*)*173 endA1 supE44 thi*-*1 recA1 gyrA96 relA1 lac* [F′ *proAB lacI*^*q*^*Z*Δ*M15* Tn*10* (Tet^r^)], obtained from Invitrogen (Carlsbad, USA), was used for cloning procedures. *E. coli* was grown at 37 °C in lysogeny broth (LB) medium supplemented with kanamycin for selection. The auxotrophic mutant *A. adeninivorans* G1212 (*aleu2 atrp1::ALEU2*), derived from *A. adeninivorans* LS3 which is deposited as *A. adeninivorans* SBUG 724 in the strain collection of the Department of Biology of the University of Greifswald, was used as the recipient strain (Kunze and Kunze 1994; Steinborn et al. 2007). *A. adeninivorans* was grown at 30 °C under nonselective conditions in complex (YDP) medium or under selective conditions in yeast minimal medium (YMM) supplemented with 2% (w/v) glucose as carbon source (Tanaka et al. 1967; Rose et al. 1990).

Fungi *T. versicolor* and *P. cinnabarinus*, which were obtained from the strain collection of the Department of Biology of the University of Greifswald and are listed there as SBUG-M1050 and SBUG-M1044 respectively, were grown on straw (inducing conditions for laccase production) at 30 °C (Herter et al. 2011).

### Isolation and heterologous expression of laccase genes

After 17 days mycelia of *T. versicolor* and *P. cinnabarinus* were harvested and total RNA was extracted using RNeasy Plus Mini Kit (Qiagen, Hilden, Germany). The cDNA was synthesized by reverse transcriptase (RevertAid Reverse Transcriptase, Thermo Fisher Scientific, Dreieich, Germany). Primers were designed based on the gene sequences taken from NCBI GenBank database (Ong et al. 1997; Otterbein et al. 2000; Benson 2004). Laccase genes with native secretion signals (GenBank Accession numbers: U44430.2, AF170093.1, U44851.1, U44431.1) were amplified by PCR using primers shown in Additional file 1: Table S1. Amplified fragments were inserted into Xplor3.2 plasmid (Bischoff et al. 2017) linearized with PacI restriction enzyme, using Gibson Assembly Master Mix [New England Biolabs (NEB), Frankfurt am Main, Germany], resulting in recombinant plasmids, which were verified by DNA sequencing. Linearization with AscI led to fragments flanked with 25S rDNA sequences (yeast rDNA integrative cassettes—YRCs) for homologous and with SbfI (yeast integrative cassettes—YICs) for non-homologous recombination. Linearized fragments were used for transformation of competent *A. adeninivorans* G1212.

To verify presence of laccase genes, genomic DNA of created strains was isolated and used as template for PCR reactions with corresponding primers listed in Additional file 1: Table S1.

Laccase gene expressing strains were preselected using 2,2′-azino-bis(3-ethylbenzothiazoline-6-sulphonic acid) (ABTS) and guaiacol agar plates. YMM plates were supplemented with 0.5 mM CuSO_4_ and 0.2 mM ABTS or 0.01% guaiacol (Soden et al. 2002; Kiiskinen et al. 2004). Appearance of green (ABTS) or red (guaiacol) halo was proof of positive transformants, which were selected and cultivated in 48-deep well plates containing YMM supplemented with 0.4 mM CuSO_4_ at 30 °C and 180 rpm. Supernatant was collected and screened for laccase activity with ABTS as substrate. Best transformant was selected for further analysis.

### Analysis of laccase gene expression

The strains *A. adeninivorans* G1212/YRC102-TEF1-TVLCC5-6H, *A. adeninivorans* G1212/YRC102-TEF1-TVLCC2-6H, *A. adeninivorans* G1212/YRC102-TEF1-TVLAC-6H, and *A. adeninivorans* G1212/YRC102-TEF1-PCLAC-6H were pre-cultivated in 10 mL YMM-NO_3_ with 2% glucose for 24 h and subsequently transferred to a second pre-culture of 50 mL YMM-NO_3_ with 2% glucose and 0.4 mM CuSO_4_. After 48 h, each strain was transferred to a 200 mL main culture of the same medium. The transfer was performed by centrifugation (4000×*g*) of the whole pre-culture and resuspension in the fresh medium. All cultivations were carried out at 30 °C and with an initial pH 5.8. Sampling occurred at 0, 2, 6, 24, 30, 48, 72, 96, and 120 h. Two pellets of each sample were obtained by centrifugation and washing with deionized water. Supernatant was collected and stored on ice for further analysis. One pellet was used for RNA extraction using RNeasy Plus Mini Kit (Qiagen, Hilden, Germany). Proteins were extracted from the second pellet using Mixer Mill MM 400 (Retsch, Haan, Germany) and McIlvaine buffer (pH 3.0). The RNA was used for cDNA synthesis with reverse transcriptase (RevertAid Reverse Transcriptase, Thermo Fisher Scientific, Dreieich, Germany). PCR using gene specific primers (Additional file 1: Table S1) was performed on cDNA to confirm transcription of each laccase gene. *AAHEXK* and *AATFIID* were used as positive control to show integrity of the cDNA.

### Enzyme activity assay

The laccase activity was determined for the oxidation of ABTS. The reaction mixture (100 µL) contained 50 mM McIlvaine buffer (pH 3.0), 3.5 mM ABTS, and 10 µL of suitably diluted sample. The appearance of green color due to ABTS oxidation was measured spectrophotometrically at 420 nm (Ɛ = 36,000 M^−1^ cm^−1^) in a Tecan Infinite M200 Microplate Reader (Tecan, Männedorf, Switzerland). In experiments where activity for 2,6-dimethoxyphenol (2,6-DMP) and syringaldazine (SGZ) was also determined, concentration of substrate in the reaction mixture was 3.5 and 0.13 mM respectively. Measurements were performed at 477 nm (Ɛ = 14,800 M^−1^ cm^−1^) for 2,6-DMP and at 530 nm (Ɛ = 65,000 M^−1^ cm^−1^) for syringaldazine. All experiments were carried out in triplicate. One unit is defined as the amount of enzyme that oxidizes 1 μmol of substrate per min under assay conditions.

### Effect of pH and temperature on activity and stability of laccase

To estimate optimal pH, laccase activity of purified protein was measured in the presence of 50 mM McIlvaine buffers with pH range 2.2–8.0. Ionic strength of buffers was adjusted to 500 mM by addition of NaNO_3_. The pH optimum was estimated for three substrates: ABTS, 2,6-DMP, and syringaldazine.

The same buffers were used for pH stability test. Enzyme was incubated at certain pH on ice and residual activity was measured after 10 min, 2 and 24 h with ABTS as substrate.

Optimal temperature was estimated by incubation of the enzyme on ice and in PCR thermocycler at temperatures ranging from 20 to 70 °C. Mixture of McIlvaine buffer (pH 3.0) and ABTS was preincubated at certain temperature for 5 min before enzyme was added. Reaction was stopped after 5 min by adding 0.625 M oxalic acid. Relative activity was measured spectrophotometrically as described above.

The effect of temperature on enzyme stability was tested by incubating the enzyme on ice and in PCR thermocycler (temperature ranging from 20 to 80 °C) and measuring residual activity after 10, 30, 60, 90, and 120 min by standard assay.

### Effect of ionic strength on laccase activity

Influence of ionic strength was estimated in presence of NaNO_3_ and NaCl. Reactions with ionic strength ranging from 0.25 to 2 M were performed and laccase activity was measured. Enzyme activity of samples with the lowest ionic strength (0.25 M) was defined as 100% and used for calculation of relative activity values.

### Determination of kinetic constants

*K*_M_ and *k*_cat_ values of purified enzyme were determined for ABTS and 2,6-DMP as substrates. Concentration of each substrate was in the range 0.5–10 mM. Enzymatic activities were measured in 50 mM citrate–phosphate buffer pH 5.0 and 25 °C. Kinetic constants of Michaelis–Menten plot were calculated using non-linear regression.

### Influence of metal ions and cofactors

The influence of different metal ions and cofactors on enzyme activity was determined by standard activity assay (described above) supplemented with 1 mM final concentration of AlCl_3_, CaCl_2_, CaSO_4_, CoCl_2_, CuCl_2_, CuSO_4_, FeCl_3_, FeSO_4_, KCl, MgCl_2_, MgSO_4_, MnCl_2_, MnSO_4_, NiCl_2_, NiSO_4_, ZnCl_2_, ZnSO_4_, or EDTA and subsequent measurement of remaining enzymatic activity. Activity assay without supplementation served as control.

### Optimization of laccase production

To investigate the influence of copper ions on laccase activity, culture medium was supplemented with CuSO_4_ in concentrations within the range of 0.2–2 mM.

To examine the influence of cultivation temperature on laccase production five Erlenmeyer flasks with YMM + NO_3_ supplemented with 0.2 mM CuSO_4_ were equally inoculated with *A. adeninivorans* G1212/YRC102-TEF1-TVLCC5-6H. Each flask was incubated at a different temperature (10, 20, 30, 37, 42 °C) for the whole experimental procedure. Yield coefficients Y_X/S_ for dry cell weight and Y_P/S_ for enzymatic activity were calculated using maximal values for biomass and activity as follows:

$$Y_{{{\raise0.7ex\hbox{$X$} \!\mathord{\left/ {\vphantom {X S}}\right.\kern-0pt} \!\lower0.7ex\hbox{$S$}}}} = \frac{{DCW_{t} - DCW_{0} }}{{Glc_{0} - Glc_{t} }}$$

$$Y_{{{\raise0.7ex\hbox{$P$} \!\mathord{\left/ {\vphantom {P S}}\right.\kern-0pt} \!\lower0.7ex\hbox{$S$}}}} = \frac{{Act_{t} - Act_{0} }}{{Glc_{0} - Glc_{t} }}$$

### Purification of Tvlcc5

Supernatant of a culture of *A. adeninivorans* G1212/YRC102-TEF1-TVLCC5-6H was harvested after 7 days of growth in YMM at 30 °C by centrifugation (4000×*g*, 5 min) and subsequently incubated with an equal volume of binding buffer (500 mM NaCl, 5 mM imidazole, 20 mM Tris–HCl pH 7.9). The mixture was loaded onto a Ni–NTA column and washed with one volume of binding buffer. After a second washing step with washing buffer containing 500 mM NaCl, 30 mM imidazole, and 20 mM Tris–HCl pH 7.9, protein was eluted with elution buffer containing 1 M imidazole. Removal of imidazole from elution fractions was performed using PD10 columns (GE Healthcare, Freiburg, Germany) and PBS buffer (phosphate-buffered saline). Purified protein was stored on ice in PBS buffer, pH 7.4 for further analysis.

### SDS-PAGE and molecular mass determination

Sodium dodecyl sulfate polyacrylamide gel electrophoresis with 10% separation gel and 4% stacking gel was carried out like described by Laemmli (1970). PageRuler Prestained Protein Ladder (Thermo Fisher Scientific, Dreieich, Germany) was used as a molecular mass marker. Protein bands were stained with InstantBlue solution (Expedeon, San Diego, USA). Western blotting was carried out by electrotransfer onto a polyvinylidene difluoride membrane. Laccase was detected using a primary polyclonal rabbit anti-6×His-tagged antibody, 200 ng mL^−1^ (MicroMol, Karlsruhe, Germany) in combination with an alkaline phosphatase-conjugated goat anti-rabbit antibody, 125 ng mL^−1^ (Promega, Mannheim, Germany). Substrate for alkaline phosphatase was nitroblue tetrazolium-5-bromo-4-chloro-3-indolylphosphate (NBT-BCIP) (Roche, Mannheim, Germany).

Native molecular mass was determined by size exclusion chromatography using a Superdex 200 column (1 by 50 cm) and 20 mM Tris–HCl buffer (pH 7.0) with 150 mM NaCl at a flow rate of 1 mL min^−1^. A mixture of ferritin (450 kDa), catalase (240 kDa), bovine serum albumin (67 kDa), and RNase A (13.7 kDa) served as molecular mass standard.

### Glycoprotein and activity staining

Glycoprotein staining was performed after SDS-PAGE development. Gels were washed 3 × 30 min in ethanol, acetic acid, water (3:1:6) solution followed by 1 h incubation in oxidizing solution (1% sodium metaperiodate in 7.5% acetic acid) in the darkness. After 5 × 30 min washing with 7.5% acetic acid, staining was carried out in fuchsin-sulfite solution (1% basic fuchsin, 1.9% sodium metabisulfite in 0.15 M HCl) for 1 h in the dark. Stained gels were washed in 1% sodium metabisulfite solution in 0.1 M HCl 5 × 30 min and afterwards 30 min in water.

Activity staining was performed using native PAGE. Enzyme bands were visualized by incubating the gel in 50 mM sodium acetate buffer pH 4.5 containing 0.5 mM ABTS or 2,6-DMP.

### Deglycosylation of Tvlcc5

*N*-linked glycans were removed from purified protein by treatment with PNGase F according to the manufacturer’s protocol (NEB, Frankfurt am Main, Germany). The product of endoglycosidase treatment was analyzed by SDS-PAGE and western blotting.

### Fed-batch fermentation

Fed-batch cultivation was carried out in a 5 L BIOSTAT Bplus TWIN (Sartorius, Göttingen, Germany) with a starting volume of 3 L medium containing 0.2 M glucose, 40 g L^−1^ peptone, 20 g L^−1^ yeast extract, 86.9 mM NH_4_H_2_PO_4_, 20.1 mM K_2_HPO_4_, 25.7 mM KH_2_PO_4_, 8.1 mM MgSO_4_·7H_2_O, 0.1% (v/v) trace elements solution, 84.7 μM Ca(NO_3_)_2_·4H_2_O, and 7.4 µM FeCl_3_·6H_2_O. The medium was supplemented with 0.4 mM CuSO_4_. Feeding was growth controlled using a custom made S88 protocol (BioPAT^®^MFCS/win 3.0, Sartorius, Göttingen, Germany). Stirrer speed was used as indirect indicator for growth via oxygen uptake. Feeding was initiated when stirrer speed dropped below 800 rpm each feeding cycle. Salt feed medium contained 1.4 mM NH_4_H_2_PO_4_, 0.2 mM MgSO_4_·7H_2_O, 6% (v/v) trace elements solution, 5.1 mM Ca(NO_3_)_2_·4H_2_O, and 0.4 mM FeCl_3_·6H_2_O. Sugar feed medium contained 50% (w/v) glucose. Feeding stocks were supplemented with 0.4 mM CuSO_4_. The C:N-ratio was kept constant during the whole feeding procedure. Fermentation was carried out at 30 °C, pH was maintained at 6.0 using 10% NaOH and 10% H_2_SO_4_. To prevent foam formation, Struktol SB 2071 (Schill + Seilacher, Böblingen, Germany) was used as antifoam agent.

### Degradation of pharmaceuticals

Degradation reactions were performed in Erlenmeyer flasks containing lyophilized Tvlcc5 (20 U mL^−1^), 50 mM sodium citrate buffer pH 5.0, and 40 mg L^−1^ tested pharmaceutical (carbamazepine, diclofenac sodium salt, sulfamethoxazole). Flasks were incubated at 30 °C on a shaker (180 rpm) for 24 h. Samples were taken after 30 min, 1, 2, 4, 8, and 24 h. Influence of laccase redox mediator on degradation process was tested by addition of 1 mM ABTS to the reaction mixture. Samples were analyzed by thin-layer chromatography.

### Thin-layer chromatography (TLC)

Prior to TLC analysis samples were extracted with 1 vol. ethyl acetate. CAMAG Automatic TLC Sampler (CAMAG, Muttenz, Switzerland) was used for sample application on pre-coated TLC sheets POLYGRAM SIL G/UV_254_ (Macherey–Nagel, Düren, Germany). Plates were developed with hexane, ethyl acetate, and acetic acid (60:40:0.1) as eluent and were analyzed with a CAMAG TLC Scanner. R_f_ values and amounts of each compound were calculated using winCATS software (CAMAG, Muttenz, Switzerland).

## Results

### Screening for laccase activity

**Fig. 1:**
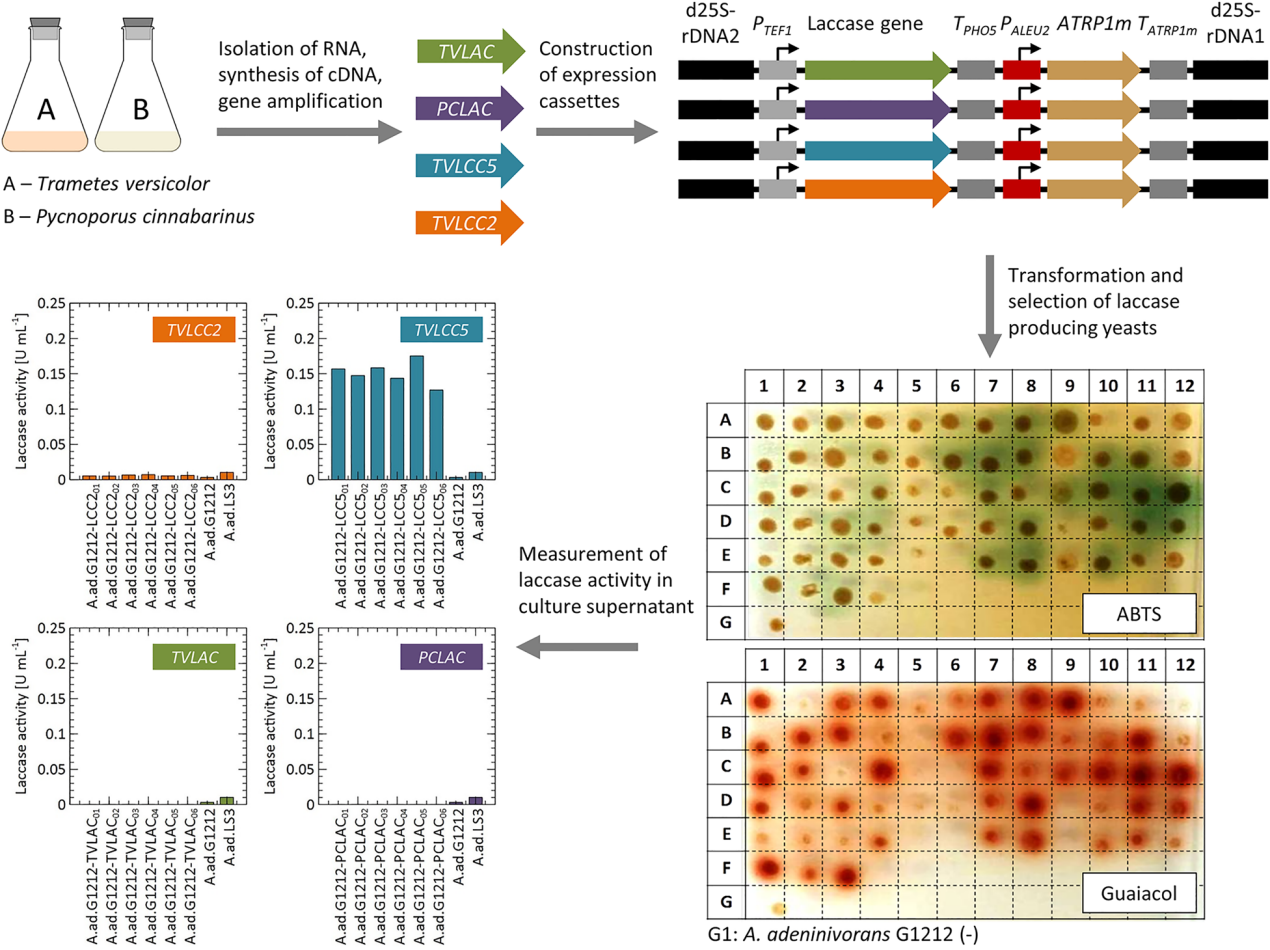
Isolation of different laccase genes from *T. versicolor* and *P. cinnabarinus* for the expression in *A. adeninivorans* G1212 using the strong constitutive *A. adeninivorans* derived *TEF1* promoter (*P*_*TEF1*_) and the *PHO5* terminator from *S. cerevisiae* (*T*_*PHO5*_). Laccase gene expressing strains were preselected using ABTS and guaiacol agar plates. For the quantification of secreted laccase, the selected strains were grown in liquid minimal medium for 3 days. Laccase activity of the supernatant was estimated after centrifugation using ABTS as substrate

For the isolation of laccase genes, the fungi *T. versicolor* and *P. cinnabarinus* were grown in YPD medium containing straw and CuSO_4_ to induce laccase gene expression. Mycelia were harvested after 17 days of cultivation and subsequently used for RNA extraction. Four ORF´s encoding for laccases were amplified via PCR (see “Materials and methods”). After construction of expression cassettes containing the strong constitutive *TEF1* promoter from *A. adeninivorans* and the *S. cerevisiae* derived *PHO5* terminator, the yeast strain *A. adeninivorans* G1212 was transformed and screened for laccase secretion using solid media with ABTS or guaiacol as substrate. Both types of transformants were screened (with homologous and non-homologous integration) and obtained results were comparable. The strains showing the strongest green or red halo were chosen for further quantification of laccase activity in liquid minimal medium (YMM) containing 0.5 mM CuSO_4_. Presence of all four laccase genes in respective strains of *A. adeninivorans* G1212 was confirmed by PCR on isolated genomic DNA using primers for laccase genes amplification (Additional file 1: Fig. S1). However, only strains containing the *TVLCC5* gene showed secretion of active laccase into the culture medium (Fig. 1). The best transformant of *A. adeninivorans* G1212/YRC102-TEF1-TVLCC5-6H was taken for further analysis.

To exclude potential dysfunctionality of the secretion system, intracellular laccase activity was measured and western blot analysis using anti-6×His antibody was performed for all four strains (Additional file 1: Fig. S2, section I and II). Obtained results indicate lack of synthesis of Tvlac, Tvlcc2, and Pclac. Within analyzed time point samples no degradation products of those proteins were identified.

For strain *A. adeninivorans* G1212/YRC102-TEF1-TVLCC5-6H distribution of active enzyme between intra- and extracellular fractions during cultivation process was analyzed (Additional file 1: Fig. S2, section III). Amount of secreted protein is increasing during cell growth, reaching the maximum of 89.4 ± 0.4% after 120 h.

Reverse transcription PCR was performed to analyse expression of *TVLAC*, *TVLCC2*, and *PCLAC*. Therefore, RNA was extracted from samples taken over the cultivation time of the described experiment (Analysis of laccase gene expression, “Materials and methods”). After cDNA synthesis, PCR was performed using primers amplifying 150 to 220 bp large fragments of a non-conserved region of each gene to eliminate false positive bands amplified from multi-copper oxidase like genes from *A. adeninivorans*. The genes for hexokinase (*AAHEXK*) and transcription initiation factor *AATFIID* were used as positive control to verify the integrity of the extracted RNA as well as to normalise the signal intensity to some extent. Additional file 1: Fig. S3a shows the amplified PCR fragments after 24 h of cultivation. Positive results were obtained for both control genes as well as *TVLCC5*, *TVLCC2*, and *PCLAC*. On the other hand, no amplification was achieved for *TVLAC* after 24 h. Additionally, it is shown that a positive signal was only obtained from the respective primers for each laccase gene, which means there is no cross signal and only one laccase gene is present in each strain. *TVLCC2* and *TVLAC* being an obvious exception since they show sequence similarity so that the same primers were used for amplification. Since no signal was detected from *TVLAC*, to verify expression a time course experiment was performed to examine for earlier or later expression than 24 h of cultivation time. Additional file 1: Fig. S3b shows that a positive signal was only detected in cDNA from samples taken after 2 h of cultivation. All other time points were negative for expression of *TVLAC*. We found a positive signal also for *PCLAC* after 2 h of cultivation, but not for *TVLCC2* and *TVLCC5* (Additional file 1: Fig. S3c).

### Expression and purification of Tvlcc5

**Fig. 2:**
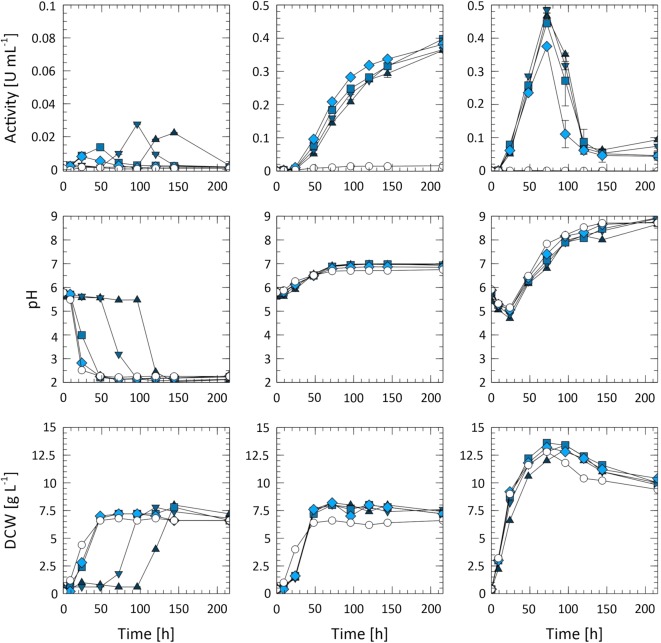
Analysis of Tvlcc5 synthesis by *A. adeninivorans* G1212/YRC102-TEF1-TVLCC5-6H in YMM-NH_4_ (left), YMM-NO_3_ (middle), and YPD (right) containing 0 mM (white circle), 0.2 mM (diamond), 0.5 mM (square), 1.0 mM (triangle down), and 2.0 mM (triangle up) CuSO_4_

**Fig. 3:**
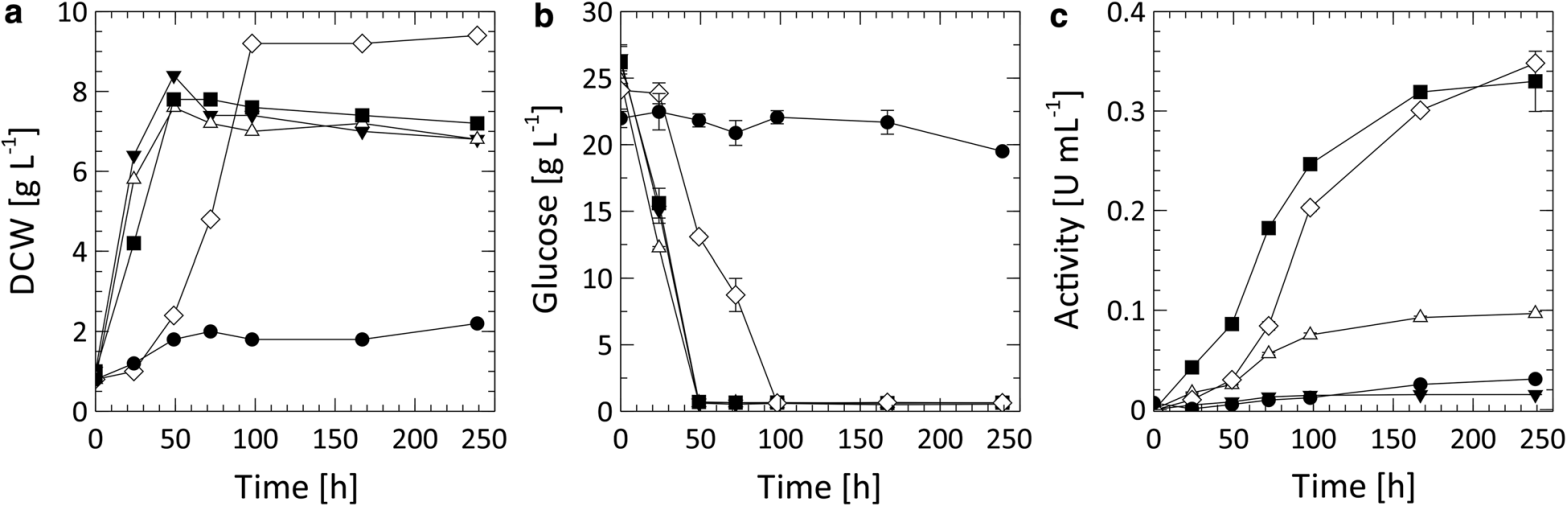
Effect of cultivation temperature on DCW (**a**), glucose concentration (**b**), and laccase activity (**c**). Strain *A. adeninivorans* G1212/YRC102-TEF1-TVLCC5-6H was grown at 10 °C (black circle), 20 °C (white diamond), 30 °C (black square), 37 °C (white up-pointing triangle), and 42 °C (black down-pointing triangle)

For purification and further analysis of Tvlcc5, it was necessary to assess the maximum activity during the cultivation. Therefore, three different media differing in the N-source were used and analyzed, concerning growth behavior and laccase secretion. Four different CuSO_4_ concentrations ranging between 0.2 and 2.0 mM were used to find the optimum for the synthesis of Tvlcc5. The results, summarized in Fig. 2 and Table 1, show that the best growth condition were found in the complex YPD medium with peptone and yeast extract as nitrogen source resulting in a yield of 0.68 ± 0.03 g_DCW_ g_glc_^−1^. The minimal media with (NH_4_)_2_SO_4_ or NaNO_3_ as nitrogen source gave comparable yields of 0.44 ± 0.04 g_DCW_ g_glc_^−1^ and 0.41 ± 0.07 g_DCW_ g_glc_^−1^, respectively. Variation in the pH value during culturing appeared to be depending on the medium used. In YPD medium the pH initially decreased from pH 6.0 to pH 4.5 after 24 h before steadily increasing until pH 9.0 after 200 h of cultivation. The most stable pH was observed in YMM-NO_3_ medium. After a gradual increase to a maximum of pH 7.0 within the first 60 h the pH remained constant for the rest of the cultivation time. The concentration of CuSO_4_ had a strong effect on both pH and growth in YMM-NH_4_ medium. The presence of NH_4_ caused an acidification of the culture medium, due to extrusion of H^+^ to maintain the necessary gradient for NH_4_-uptake (Peña et al. 1987). This caused the pH drop from pH 6.0 to 2.0 within 48 h in the presence of 0, 0.2, and 0.5 mM CuSO_4_, after 100 h in the presence of 1 mM CuSO_4_, and after 150 h in the presence of 2 mM CuSO_4_ (Fig. 2 middle left). A similar time delay was observed for reaching maximum dry cell weight in this medium (Fig. 2 down left). The highest activity of extracellular Tvlcc5 was obtained in YPD medium with an absolute peak after 60 h of incubation. After this, activity decreased due to laccase degradation in the increasingly alkaline environment. This possibly is a result of utilization of amino acids after depletion of glucose and the subsequent release of ammonia (Vylkova et al. 2011). In YMM-NO_3_ an activity of 0.4 U mL^−1^ was measured after 200 h. Tvcc5 remained stable over the full period of cultivation. In YMM-NH_4_ only a low accumulation of Tvlcc5 was detected. In the presence of all CuSO_4_ concentrations tested, maximum Tvlcc5 activity always concurred with maximum growth rate and medium attaining pH 2.0. After this peak Tvlcc5 activity decreased to 0.0 U mL^−1^, implying complete enzyme degradation (Fig. 2 top left). Additionally, no laccase activity was detected in media without the supplementation of CuSO_4_. Maximum accumulation of laccase was achieved with 0.5 mM CuSO_4_ in YMM-NO_3_ as well as in YPD. Higher concentrations of Cu^2+^ had no beneficial effect on laccase activity. To verify the influence of cultivation temperature on laccase production, cultures were grown at five different temperatures: 10, 20, 30, 37, and 42 °C (Fig. 3). Cultures grown at 30, 37, and 42 °C showed a similar biomass production profile. Cultures grown at 20 °C showed a longer lag phase but final biomass accumulation was highest. Maximum laccase activity (0.35 U mL^−1^) was also detected at this temperature with only slightly lower levels of Tvlcc5 secretion (0.33 U mL^−1^) measured at 30 °C. Incubation at 10 °C strongly inhibited growth causing low accumulation of laccase. A similarly low amount of Tvlcc5 was also detected in the 42 °C culture, which did show better cell growth compared to the 10 °C culture.

**Table 1:** Summary of the specific yields obtained during the analysis of Tvlcc5 synthesis by *A. adeninivorans* G1212/YRC102-TEF1-TVLCC5-6H in YMM-NH_4_ (NH_4_), YMM-NO_3_ (NO_3_) and YPD

Cu^2+^ (mM)	Y_X/S_ (g_DCW_ g_glc_^−1^)			Y_P/S_ (mU g_glc_^−1^)			Max. activity (mU mL^−1^)		
	NH_4_	NO_3_	YPD	NH_4_	NO_3_	YPD	NH_4_	NO_3_	YPD
0.00	0.45	0.33	0.66	0.07	0.82	0.08	1.55	15.86	0.85
0.20	0.42	0.36	0.66	1.01	20.24	25.03	7.97	381.63	375.11
0.50	0.49	0.43	0.72	1.01	24.97	34.13	13.60	397.57	444.74
1.00	0.45	0.49	0.64	1.86	24.78	32.64	27.65	365.48	484.70
2.00	0.38	0.45	0.70	1.28	23.76	35.37	22.35	363.31	467.01

The enzyme yield Y_P/S_ was calculated for the time point on which maximum activity was measured during the cultivation

**Fig. 4:**
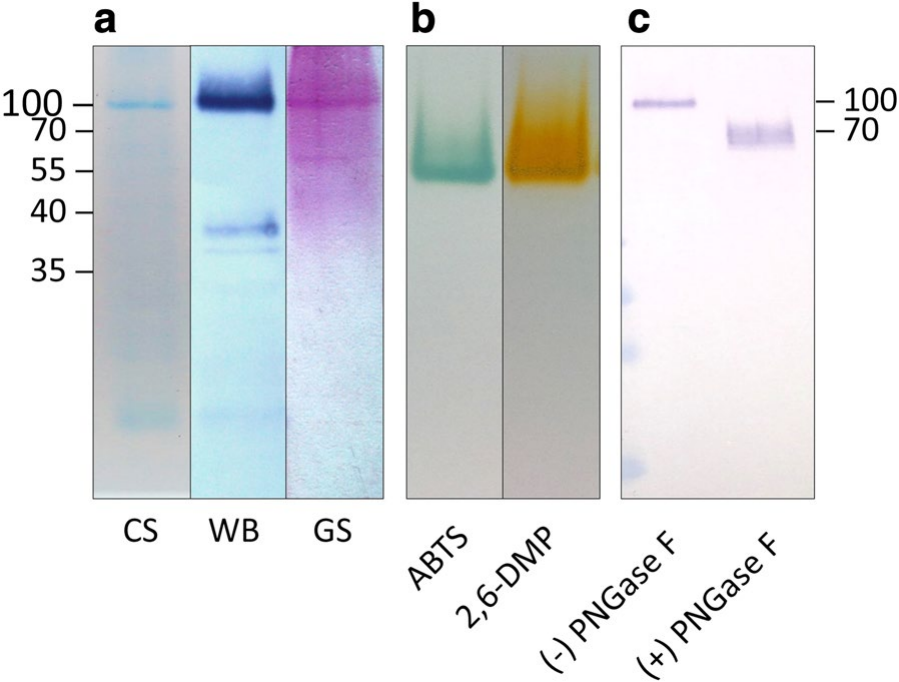
PAGE analysis of purified Tvlcc5 synthesized in *A. adeninivorans* G1212/YRC102-TEF1-TVLCC5-6H. **a** Western-blot signal (WB), Coomassie (CS) and glycosylation staining (GS) of Tvlcc5 after separation on a 10% SDS-PAA gel. **b** Activity staining of Tvlcc5 separated on a clear native gel using ABTS or 2,6-DMP as substrates (no marker was used since separation in native gels is by size and charge simultaneously). **c** Comparison of the western-blot signals of Tvlcc5 and PNGase F treated Tvlcc5

For the biochemical characterization, it was necessary to purify Tvlcc5 to homogeneity. Therefore, 4 L of YMM-NO_3_ were inoculated with *A. adeninivorans* G1212/YRC102-TEF1-TVLCC5-6H and cultivated for 7 days. The culture supernatant was mixed with one volume of binding buffer leading to a drop in activity from an initial 0.358 ± 0.013 U mL^−1^ down to 0.016 ± 0.004 U mL^−1^ due to the presence of Cl^−^ ions (500 mM NaCl, as described in “Materials and methods”). Final activities of 0.882 ± 0.041, 0.589 ± 0.043, and 0.326 ± 0.016 U mL^−1^ were recovered in three subsequent elution steps. SDS-PAGE analysis of the Tvlcc5 elution steps showed a band at around 100 kDa (theoretical molecular mass 53.6 kDa), which was confirmed by western blotting to be a His-tagged protein (Fig. 4a, lane 2). Size exclusion chromatography in native conditions revealed a value of 90 kDa, thus corresponding to the result of the SDS-PAGE analysis. Since more than one band was visible after Coomassie staining, a clear native gel was performed to confirm that no other protein with laccase activity was present in the elution fraction. The activity staining with ABTS and 2,6-DMP (Fig. 4b) clearly showed a single band only, indicating sufficient purity for further characterization. The discrepancy between the theoretical molecular mass of 53.6 kDa and the measured value of 100 kDa might be due to hyperglycosylation. Both, staining for glycosylation (Fig. 4a, lane 3) as well as altered SDS-PAGE location after deglycosylation by PNGase F (Fig. 4c) confirmed that Tvlcc5 is highly glycosylated.

### Biochemical characterization

The biochemical parameters of purified Tvlcc5 were analyzed in detail to determine the optimal reaction conditions for its application in the degradation of pharmaceuticals.

**Fig. 5:**
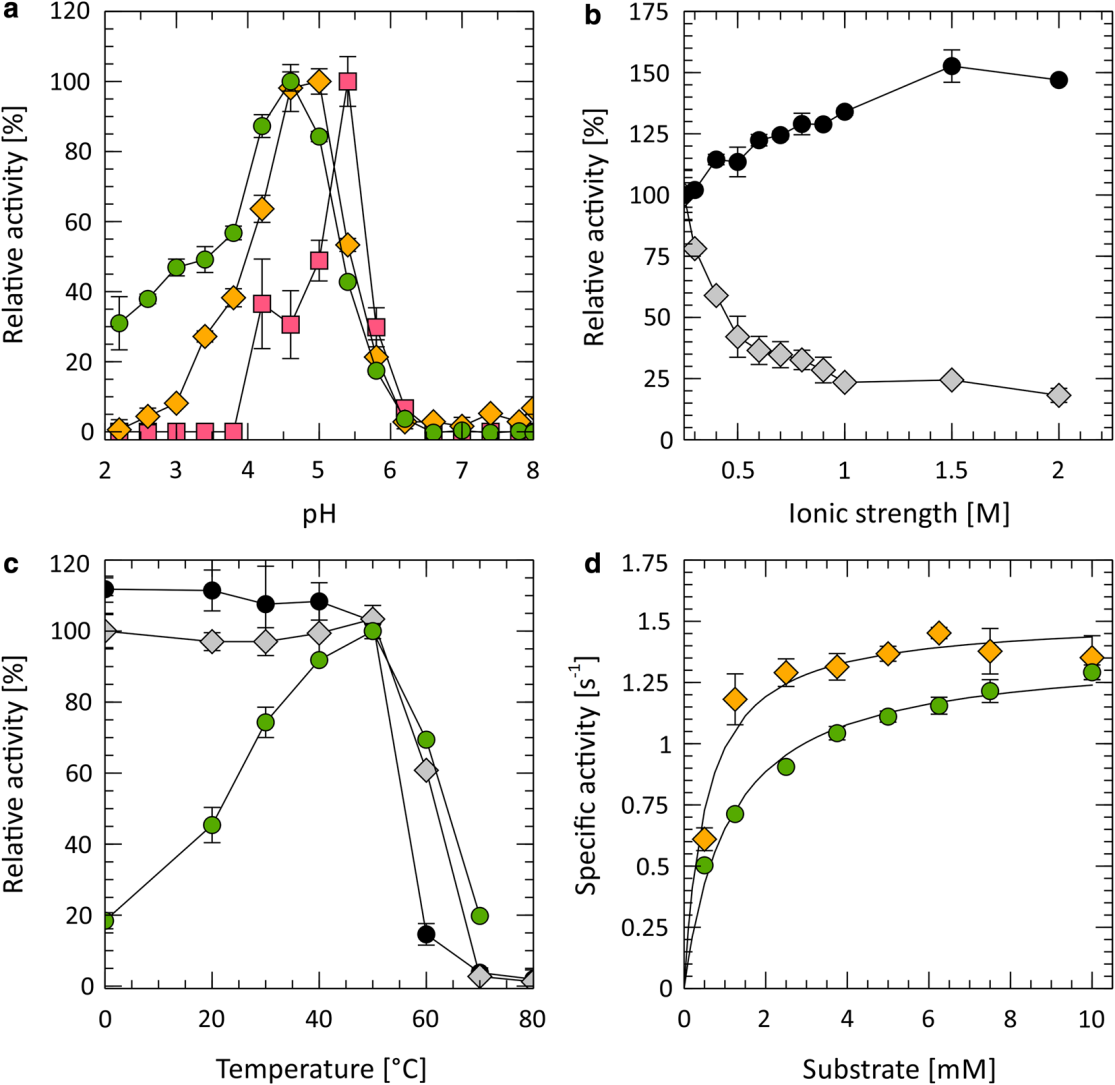
**a** Effect of pH on the activity of purified Tvlcc5 with ABTS (green circle), 2,6-DMP (yellow diamond), and SGZ (pink square) as substrate. Activity was calculated in relation to the maximum value for each substrate. **b** Influence of ionic strength on the activity of purified Tvlcc5. Ionic strength was adjusted using NaNO_3_ (black circle) or NaCl (grey diamond). The activity in pure buffer without addition of any salt was set to 100%. **c** Effect of temperature (green circle) on the activity of Tvlcc5 and residual activity after incubation at various temperatures for 10 (grey diamond) and 60 min (black circle). **d** Influence of the concentration of ABTS (green circle) and 2,6-DMP (yellow diamond) on the specific activity of purified Tvlcc5

The optimal pH for Tvlcc5 was between pH 4.5 and 5.0 for the substrates ABTS and 2,6-DMP. In case of SGZ as substrate, the highest relative activity is pH 5.5 (Fig. 5a). The ionic strength of the reaction buffer was found to be a key factor influencing the pH optimum. Without correction of ionic strength to 0.5 M, the pH optimum for the three substrates used in this experiment varied from pH 2.6 for ABTS, pH 3.8 for 2,6-DMP to pH 4.6 for SGZ (Additional file 1: Fig. S4). Stability of Tvlcc5 was tested by incubation for 2 or 24 h under various pH regimes in the presence of buffers with equalized ionic strength. Enzyme instability in acidic conditions measured in loss of initial activity was most extreme at pH 2.2. Under this condition, activity decreased to 61.0 ± 6.8% after 2 h and was down to 22.7 ± 7.7% after 24 h (data not shown). Substantial drops in activity were also measured at pH 3.4 after 2 h (76.6 ± 17.7%) and after 24 h (60.1 ± 12.6%).

Tvlcc5 activity also depends on the ionic strength of the reaction buffer. Figure 5b shows this relationship for NaNO_3_ and NaCl, which were used to adjust the ionic strength as described in “Materials and methods”. Increasing ionic strength to 2 M using NaNO_3_ led to increase of relative laccase activity to 147.0 ± 0.7%. On the other hand, using NaCl led to decrease of activity to 18.2 ± 2.9% of initial value.

The relative activity of Tvlcc5 initially increased with temperatures until 50 °C, before decreasing due to its instability above 55 °C (Fig. 5c). The results show a temperature optimum between 35 and 55 °C with a maximum activity at 50 °C. Lower activities of 18.4 and 19.8% were achieved by performing the reaction either on ice or at 70 °C respectively. The half-life of Tvlcc5 at 60 °C was calculated to be 20.94 min.

Parameters of Michaelis–Menten kinetics were calculated with non-linear regression for ABTS and 2,6-DMP as substrates. Values of Michaelis constant, turnover number, and catalytic efficiency (specificity constant) for each substrate are summarized in Table 2. The Michaelis constant (*K*_M_) for ABTS is twofold higher than for 2,6-DMP, which indicates that the concentration of ABTS has to be higher to reach maximum velocity. Both substrates have similar turnover numbers (*k*_cat_), but 2,6-DMP has a higher specificity constant (*k*_cat_ *K*_M_^−1^) indicating that this substrate is preferably used by Tvlcc5.

**Table 2:** Michaelis–Menten kinetic constants of purified Tvlcc5

	ABTS	2,6-DMP
*K*_M_ (mM)	1.1 ± 0.1	0.5 ± 0.1
*k*_cat_ (s^−1^)	1.4 ± 0.0	1.5 ± 0.0
*k*_cat_*K*_M_^−1^ (mM^−1^s^−1^)	1243.5 ± 291.4	2808.3 ± 473.6

Enzymatic activities were measured in 50 mM citrate–phosphate buffer pH 5.0 and 25 °C

Chloride and sulfate salts of a range of metals were tested for their ability to influence Tvlcc5 activity. The results summarized in Table 3 show that in most cases activity decreased in the presence of chloride salts, whereas in the presence of sulfate salts activity remained stationary or slightly increased. This indicates that most metal ions have no effect on Tvlcc5 activity, with Fe being the only exception. Both FeCl_3_ and FeSO_4_ caused a strong reduction in enzyme activity indicating an overall negative influence of iron ions.

**Table 3:** Tvlcc5 activity in the presence of metal salts (1 mM)

Addition of:	Activity [%]	Addition of:	Activity [%]
CaCl_2_	51.9 ± 0.8	CaSO_4_	113.9 ± 7.3
CuCl_2_	48.3 ± 2.3	CuSO_4_	111.1 ± 3.1
FeCl_3_	22.1 ± 11.8	FeSO_4_	0.0 ± 4.9
MgCl_2_	49.0 ± 6.7	MgSO_4_	106.0 ± 0.9
MnCl_2_	30.5 ± 3.1	MnSO_4_	108.9 ± 1.6
NiCl_2_	39.0 ± 5.8	NiSO_4_	99.3 ± 3.0
ZnCl_2_	38.5 ± 2.7	ZnSO_4_	104.9 ± 0.7
AlCl_3_	32.2 ± 1.7	EDTA	116.6 ± 2.3
CoCl_2_	44.0 ± 1.3	(–)	100.0 ± 2.3
KCl	65.5 ± 4.1		

### Fed-batch cultivation

**Fig. 6:**
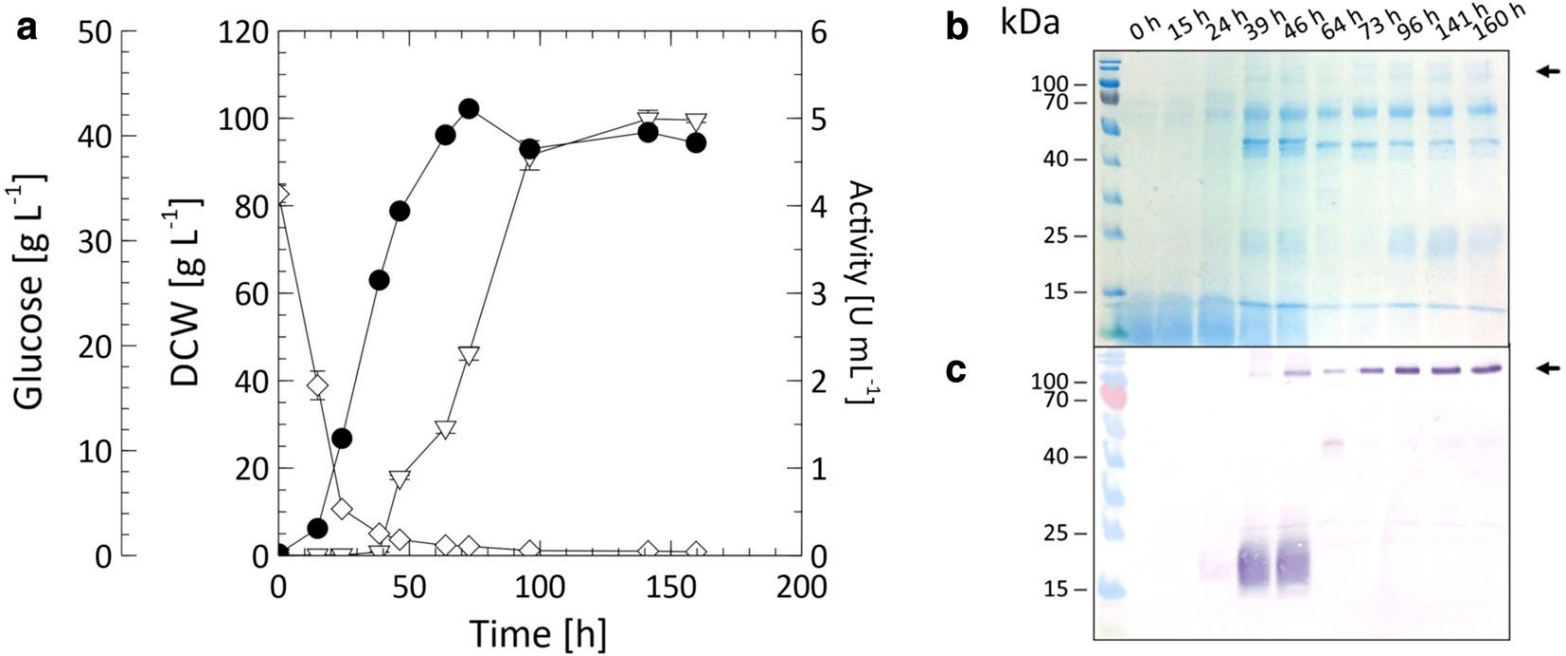
**a** Time course of DCW (black circle), glucose concentration (white diamond), and laccase activity (white up-pointing triangle) during a representative fed-batch cultivation of *A. adeninivorans* G1212/YRC102-TEF1-TVLCC5-6H. Coomassie-stained SDS-PAGE (**b**) and western-blot analysis with anti-6×His antibody **c** show the accumulation of recombinant Tvlcc5 in the supernatant of samples taken at each time point

A lab-scale cultivation of *A. adeninivorans* G1212/YRC102-TEF1-TVLCC5-6H was required to obtain enough enzyme to study the Tvlcc5 degradation of pharmaceuticals. After the initial glucose was utilized to a residual concentration of 4.44 ± 0.16 g L^−1^, automated and growth-controlled feeding was initialized. The cultivation was concluded after 160 h with a final dry cell weight of 96.52 ± 3.51 g L^−1^ and an extracellular laccase activity of 4986.3 U L^−1^ resulting in yields of 51.66 U g_DCW_^−1^, 37.2 U g_glc_^−1^, and 0.72 g_DCW_ g_glc_^−1^. The time course of dry cell weight, glucose concentration and Tvlcc5 activity are shown in Fig. 6.

### Degradation of carbamazepine, diclofenac, and sulfamethoxazole

**Fig. 7:**
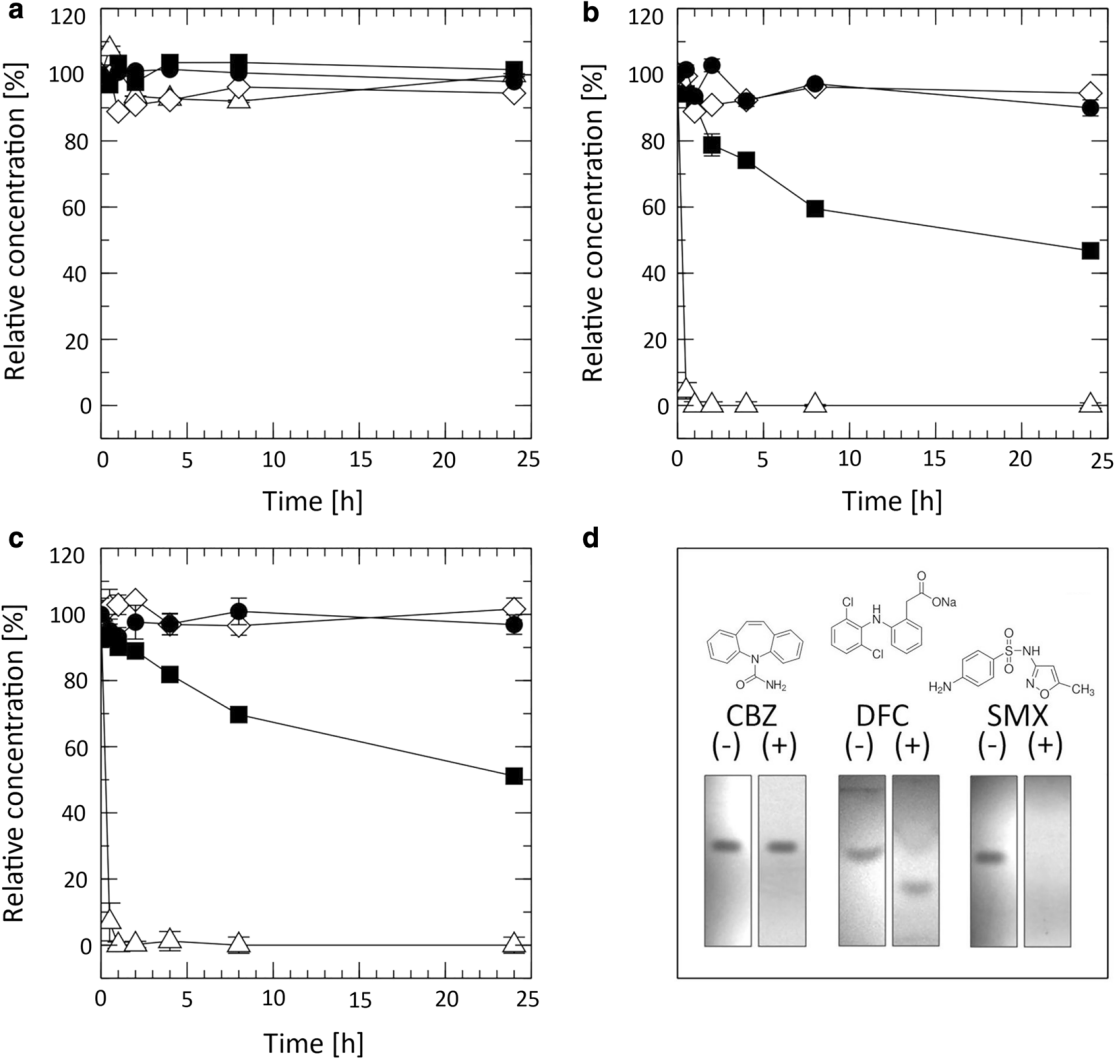
Degradation courses of carbamazepine (**a**), diclofenac (**b**), and sulfamethoxazole (**c**). Reaction mixtures containing pharmaceutical and Tvlcc5 in the absence (black square) or presence of 1 mM ABTS (white up-pointing triangle) were compared with reaction mixtures containing pharmaceuticals only in the absence (black circle) or presence of 1 mM ABTS (white diamond). **d** Thin layer chromatography plates showing influence of ABTS (+) on degradation of pharmaceuticals

Investigations on Tvlcc5 laccase mediated degradation of three common pharmaceutical pollutants of wastewater (carbamazepine, diclofenac, and sulfamethoxazole) (Sacher et al. 2008; European Environment Agency 2010) showed significant effects in case of diclofenac (46.8% breakdown) and sulfamethoxazole (51.1% breakdown), whereas carbamazepine remained virtually unaffected (Fig. 7a–c). Addition of the laccase mediator ABTS significantly increased the efficiency of Tvlcc5. Under these conditions, diclofenac and sulfamethoxazole were fully degraded within 1 h of incubation. Carbamazepine, on the other hand, remained unaffected (Fig. 7d).

## Discussion

Of the four laccase genes isolated from the fungi *T. versicolor* and *P. cinnabarinus*, only *TVLCC5* was successfully expressed in the host yeast *A. adeninivorans*. Although successful gene insertion (Additional file 1: Fig. S1) as well as transcription were confirmed for all laccase genes (Additional file 1: Fig. S3), no other than *TVLCC5* leads to detectable protein neither in supernatant nor within the cells. Even while using the same promoter-terminator combination, the strains showed different transcription patterns. *TVLAC* and *PCLAC* were already detectable after 2 h of cultivation, whereas signals for mRNA of *TVLCC5* and *TVLCC2* were first found in cDNA from samples taken after 24 h. Furthermore, degradation of mRNA from *TVLAC* seems to occur at a high rate, since no signal was detected anymore after 2 h of cultivation. This leads to the assumption that either the transcripts for *TVLCC2*, *TVLAC*, and *PCLAC* are not translated at all, which could be caused by rapid degradation and issues with secondary structure, or they are translated but the freshly formed protein is immediately degraded due to putative protease recognition sites. By means of multiple sequence alignment (Additional file 1: Fig. S5) we identified six domains with amino acid sequence differences that may be related to this problem. The identified sequences show a high degree of similarity or are even identical between Tvlac, Tvlcc2, and Pclac. The amino acid sequence of Tvlcc5, on the other hand, differs in those positions, e.g., 49–51 LAG instead of VVN (47–49 Tvlac and Tvlcc2; 48–50 Pclac), 253–255 HEA instead of SQP/TQP (248–250 Tvlac and Tvlcc2; 249–251 Pclac), 294–296 DTT instead of GFA (289–291 Tvlac and Tvlcc2; 290–292 Pclac), 308–310 TAE instead of GAP (303–305 Tvlac and Tvlcc2; 304–306 Pclac), 346–348 PAA instead of MPV (341–343 Tvlac and Tvlcc2; 342–344 Pclac) or 433–435 TFS (428–430 Tvlac and Tvlcc2; 429–431 Pclac) instead of AFA (Additional file 1: Fig. S5). Furthermore, the domain IITTD in position 411–415 of Tvlcc5, which is absent from Tvlcc2, Tvlac and Pclac, could be involved in post translational modifications. The absence of this sequence could be another cause for the early degradation of the three proteins. Post-translational modifications are often species specific and failures can lead to incorrect folding by chaperones, different pattern of glycosylation, phosphorylation, or disulfide bonds formation during recombinant protein production (Tokmakov et al. 2012; Kim et al. 2015). It is conceivable that *A. adeninivorans* is unable to process those modifications for different proteins with the same efficiency, leading to the production of non-functional proteins prone to degradation. Issues with codon usage bias, on the other hand, are more unlikely since an analysis of the codon-usage showed that all four laccase genes show some rare codons, but none of them would be excluded from being translated by *A. adeninivorans*. The distribution of Tvlcc5 between intra- and extracellular space as well as the signals of protein degradation products indicate an ineffective secretion of this enzyme by the host. Since we used the native signal sequence in this study, a closer investigation into yeast signal peptides may be worthwhile. The use of both native laccase and host-originated signal peptides has been reported previously (Jönsson et al. 1997; Brown et al. 2002; Piscitelli et al. 2005). Results differ for each enzyme and host organism, leading to the conclusion, that choice of the most efficient signal peptide is not easy to predict and must be assayed for each expression system separately.

Determination of the molecular mass of native Tvlcc5 yielded a value of 100 kDa which is significantly larger than the 53.6 kDa predicted from the amino acid sequence. Screening of the PROSITE database (de Castro et al. 2006) for functional sites, showed that *T. versicolor* lcc5 has 13 potential *N*-glycosylation sites. Removal of *N*-glycans by treatment with PNGase F led to a marked decrease in molecular mass to approx. 70 kDa (Fig. 4). Although this is still higher than the predicted value, the remaining difference could be due to the presence of additional *O*-glycans. Hyperglycosylation by yeasts is a well-known phenomenon and would be the natural explanation for the substantial differences in molecular mass observed (Bulter et al. 2003; Piscitelli et al. 2010; Bischoff et al. 2017). The laccases produced by fungi are de facto glycoproteins that usually have a carbohydrate content of 10–20% (Thurston 1994; Morozova et al. 2007).

The optimal pH conditions for Tvlcc5 were within the range 4.5–5.5 for all tested substrates (ABTS, 2,6-DMP, and SGZ). This apparently contradicts many previous reports, showing a variation in pH optimum depending on which substrate is used (Nitheranont et al. 2011; Gu et al. 2014; Kalyani et al. 2015). We found the ionic strength of the buffer to be a key factor for this discrepancy. Without adjusting the ionic strength, the pH optimum differs for each substrate (pH 2.6 for ABTS, pH 3.8 for 2,6-DMP, and pH 4.6 for SGZ). Whereas SO_4_^2−^ salts had no or a slightly benign effect on Tvlcc5 activity, similar concentration of chloride ions was detrimental for Tvlcc5 activity decreasing enzyme activity by 50% or more (Table 3). Laccase inhibition by Cl^−^ and other halides had been already observed by Koudelka and Ettinger (1988), Xu (1996) and Garzillo et al. (1998). However, in case of iron salts, both chloride and sulfate compounds inhibited enzyme activity almost completely indicating that Fe ions substantially influence Tvlcc5 activity as well (Table 3).

Additional evidence for negative influence of Cl^−^ on laccase activity is shown in Fig. 5b. By increasing the ionic strength of the buffer, concentration of Cl^−^ is also increasing, leading to inhibition of laccase activity (18.2 ± 2.9% of the initial value after increase of ionic strength to 2 M).

Preceding large-scale production of Tvlcc5 optimization procedures were investigated. Different cultivation media were implemented. Cultivation in YPD medium yielded the highest enzyme accumulation (Fig. 2). However, in future during the cultivation in YPD the pH would have to be regulated to prevent enzyme inactivation. Medium supplementation with different amounts of CuSO_4_ showed that addition of Cu^2+^ ions is essential for enzyme activity. Supplementation with 0.5 mM CuSO_4_ was found to be optimal (Fig. 2).

In case of temperature, both cell growth and laccase activity reached optimal levels at 20 °C (9.4 g L^−1^, 0.35 U mL^−1^, respectively) (Fig. 3). Laccase accumulation was only slightly lower at 30 °C (0.33 U mL^−1^), but showed a marked decline under higher temperatures. This may be due to the temperature-dependent dimorphism of *A. adeninivorans*, which at 42 °C transitions from budding cells into mycelia (Wartmann et al. 1995).

A fed-batch cultivation of *A. adeninivorans* G1212/YRC102-TEF1-TVLCC5-6H achieved a laccase production of 4986.3 U L^−1^ (Fig. 6), which is more than three-fold higher than obtained by Brown et al. (2002) using *P. pastoris* for the production of the same isozyme from *T. versicolor*. Phenols are the typical natural substrates for laccases. However, under certain conditions laccases may also oxidize non-phenolic substrates. The oxidation reaction can be enhanced by addition of appropriate mediators (Bourbonnais and Paice 1990; Baiocco et al. 2003; Cañas and Camarero 2010). The pharmaceuticals tested in this study are non-phenolic substrates. We found the redox mediator ABTS to play a key role in accelerating the degradation of diclofenac and sulfamethoxazole, though it had no influence on the degradation of carbamazepine. To address this problem, an alternative laccase-mediator system may be necessary (Baiocco et al. 2003). Considering environmental and economic aspects natural mediators should be preferred. Future work will concentrate on generating a stable yeast strain, optimized for both laccase and cytochrome P450 production, which can be applied as a whole-cell catalyst for the deactivation of active pharmaceutical compounds in wastewater treatment plants. An important issue to solve in the future is the distribution of laccase. Rather than it being secreted into the environment it would be optimal to locally concentrate this enzyme by attachment to the cell surface. Another option would be the co-immobilization of enzyme with yeast cells.

## Additional file

**Additional file 1.** **Table S1.** Sequences of primers for gene amplification and reverse transcription PCR. **Fig. S1.** PCR on genomic DNA of *A. adeninivorans* G1212 strains expressing laccase genes using primers for gene amplification. As negative controls the same PCR reactions were performed using genomic DNA of *A. adeninivorans* G1212(-) as template. Thermo Scientific O’GeneRuler 1 kb Plus served as DNA ladder. **Fig. S2.** (**I**) Intracellular (upper row) and extracellular (lower row) laccase activity of (**a**) *A. adeninivorans* G1212 (negative control) and strains expressing (**b**) *TVLCC5*, (**c**)*TVLCC2*, (**d**) *TVLAC*, (**e**) *PCLAC* genes. (**II**) Western-blot and Coomassie-stained SDS-PAGE analysis of intracellular (1, 3) and extracellular (2, 4) fractions isolated after 48 (1, 2) and 120 h (3, 4) of cultivation. (**III**) Distribution of Tvlcc5 protein between intracellular (dark grey) and extracellular (light grey) fraction during 120 h cultivation of *A. adeninivorans* G1212/YRC102-TEF1-TVLCC5-6H. **Fig. S3.** (**a**) RT-PCR on samples taken from *A. adeninivorans* G1212/YRC102-TVLCC5-6H, *A. adeninivorans* G1212/YRC102-TVLCC2-6H, *A. adeninivorans* G1212/YRC102-TVLAC-6H, *A. adeninivorans* G1212/YRC102-PCLAC-6H, and *A. adeninivorans* G1212/YRC102 after 24 h of cultivation for amplification of fragments of *TVLCC5* (1), *TVLAC* (2), *PCLAC* (3), *AAHEXK* (H), and *AATFIID* (T) using primers shown in Table S1. (**b**) RT-PCR on samples taken from *A. adeninivorans* G1212/YRC102-TVLAC-6H after the indicated cultivation time for amplification of a fragment of *TVLAC*. (**c**) RT-PCR on samples taken from the same cultivations after 2 h for amplification of fragments of *TVLCC5* (1), *TVLAC* (2), *PCLAC* (3), *AAHEXK* (H), and *AATFIID* (T) using primers shown in Table S1. **Fig. S4.** Effect of pH on the activity of purified Tvlcc5 with ABTS (green circle), 2,6-DMP (yellow triangle), and SGZ (pink square) as substrate in presence of buffers without equalized ionic strength. Activity was calculated in relation to the maximum value for each substrate. **Fig. S5.** Alignment of the protein sequences derived from *TVLCC5*, *TVLAC*, *TVLCC2*, and *PCLAC* genes in box shade format. Black box shows identical amino acids. Grey box implies amino acids with similar residues. White text indicates amino acids without similarities.

## Data Availability

The data sets supporting the conclusions of this article are included within the article. Further data (named data not shown in the manuscript) are available from the corresponding author on reasonable request.

## Abbreviations

ABTS: 2,2′-azino-bis(3-ethylbenzothiazoline-6-sulphonic acid)
YRC: yeast rDNA integrative expression cassette
YIC: yeast integrative expression cassette
2,6-DMP: 2,6-dimethoxyphenol
SGZ: syringaldazine
DCW: dry cell weight
