# Supplementary material for: Characterization of recombinant laccase from Trametes versicolor synthesized by Arxula adeninivorans and its application in the degradation of pharmaceuticals
Source: AMB Express. 2019 Jul 11;9:102. doi: 10.1186/s13568-019-0832-3 (PMC6624219; doi:10.1186/s13568-019-0832-3)
Supplement: Supplementary file 1 — Additional file 1. Table S1. Sequences of primers for gene amplification and reverse transcription PCR. Fig. S1. PCR on genomic DNA of A. adeninivorans G1212 strains expressing laccase genes using primers for gene amplification. As negative controls the same PCR reactions were performed using genomic DNA of A. adeninivorans G1212(-) as template. Thermo Scientific O’GeneRuler 1 kb Plus served as DNA ladder. Fig. S2. (I) Intracellular (upper row) and extracellular (lower row) laccase activity of (a) A. adeninivorans G1212 (negative control) and strains expressing (b) TVLCC5, (c)TVLCC2, (d) TVLAC, (e) PCLAC genes. (II) Western-blot and Coomassie-stained SDS-PAGE analysis of intracellular (1, 3) and extracellular (2, 4) fractions isolated after 48 (1, 2) and 120 h (3, 4) of cultivation. (III) Distribution of Tvlcc5 protein between intracellular (dark grey) and extracellular (light grey) fraction during 120 h cultivation of A. adeninivorans G1212/YRC102-TEF1-TVLCC5-6H. Fig. S3. (a) RT-PCR on samples taken from A. adeninivorans G1212/YRC102-TVLCC5-6H, A. adeninivorans G1212/YRC102-TVLCC2-6H, A. adeninivorans G1212/YRC102-TVLAC-6H, A. adeninivorans G1212/YRC102-PCLAC-6H, and A. adeninivorans G1212/YRC102 after 24 h of cultivation for amplification of fragments of TVLCC5 (1), TVLAC (2), PCLAC (3), AAHEXK (H), and AATFIID (T) using primers shown in Table S1. (b) RT-PCR on samples taken from A. adeninivorans G1212/YRC102-TVLAC-6H after the indicated cultivation time for amplification of a fragment of TVLAC. (c) RT-PCR on samples taken from the same cultivations after 2 h for amplification of fragments of TVLCC5 (1), TVLAC (2), PCLAC (3), AAHEXK (H), and AATFIID (T) using primers shown in Table S1. Fig. S4. Effect of pH on the activity of purified Tvlcc5 with ABTS (green circle), 2,6-DMP (yellow triangle), and SGZ (pink square) as substrate in presence of buffers without equalized ionic strength. Activity was calculated in relation to the maximum value for each substrate. Fig. [file 13568_2019_832_MOESM1_ESM.pdf]

AMB Express

Characterization of recombinant laccase from *Trametes versicolor* synthesized by *Arxula adeninivorans* and its application in the degradation of pharmaceuticals

Katarzyna Litwińska[a], Felix Bischoff[a,b], Falko Matthes[a], Rüdiger Bode[c], Twan Rutten[a], Gotthard Kunze[a]

[a] Leibniz Institute of Plant Genetics and Crop Plant Research (IPK), Corrensstr. 3, 06466 Gatersleben, Germany

[b]Jäckering Mühlen- und Nährmittelwerke GmbH, Vorsterhauser Weg 46, 59007 Hamm, Germany

[c] Institute of Microbiology, University of Greifswald, Jahnstr. 15, 17487 Greifswald, Germany

*Corresponding author: G. Kunze, Leibniz Institute of Plant Genetics and Crop Plant Research (IPK), Corrensstr. 3, 06466 Gatersleben, Germany, e-mail: kunzeg@ipk-gatersleben.de, phone: +49 (0)39482 5247

Table S1 Sequences of primers for gene amplification and reverse transcriptase PCR

Gene amplification			
Gene [GenBank Accession number]	Primer sequence (5‘ → 3‘)[a,b]	Direction	Plasmid
TVLAC [U44430.2] (Ong et al. 1997)	TAAAAACATAATCAACATTAATATGGGTCTGCAGCGATTCAG	fwd	Xplor3.2-TEF1- TVLAC(-6H)-PHO5
	TAATGAGTAGTGTTCCTTAATTCACTGGTTAGCCTCGC	rev	
	TAATGAGTAGTGTTCCTTAATTCA**GTGGTGGTGATGATGGTG**CTGGTTAGCCTCGC	rev	
PCLAC [AF170093.1] (Otterbein et al. 2000)	TAAAAACATAATCAACATTAATATGTCGAGGTTCCAGTC	fwd	Xplor3.2-TEF1- PCLAC(-6H)-PHO5
	TAATGAGTAGTGTTCCTTAATTCAGAGGTCGCTGGGGTCAAGTG	rev	
	TAATGAGTAGTGTTCCTTAATTCA**GTGGTGGTGATGATGGTG**GAGGTCGCTGGGGTCAAGTG	rev	
TVLCC2 [U44851.1]	TAAAAACATAATCAACATTAATATGGGTCTGCAGCGATTC	fwd	Xplor3.2-TEF1- TVLCC2(-6H)-PHO5
	TAATGAGTAGTGTTCCTTAATTCATTGGTTTGCCTCG	rev	
	TAATGAGTAGTGTTCCTTAATTCA**GTGGTGGTGATGATGGTG**TTGGTTTGCCTCG	rev	
TVLCC5 [U44431.1] (Ong et al. 1997)	TAAAAACATAATCAACATTAATATGGGCAAGTTTCACTC	fwd	Xplor3.2-TEF1- TVLCC5(-6H)-PHO5
	TAATGAGTAGTGTTCCTTAATTCAGAGGTCGGACGAG	rev	
	TAATGAGTAGTGTTCCTTAATTCA**GTGGTGGTGATGATGGTG**GAGGTCGGACGAG	rev	
**Reverse transcriptase PCR**			
Fragment (size)	Primer sequence (5‘ → 3‘)	Direction	
**TVLCC5** (151 bp)	CTCCGTCATCCCTCTTAC	fwd	
	GACGAAGGTCTCGTTGTT	rev	
**TVLAC/TVLCC2** (180 bp)	AGTCGCCGAGCCCACTAC	fwd	
	GTGAAAGTCGCGTTGTTG	rev	

**PCLAC** (184 bp)	ACCCGAGATCGAGCCTAC	fwd
	GACAAAGGTGTGGTCGTTG	rev
**AAHEXK** (219 bp)	TTGACCGAGGTTGAGCTTCT	fwd
	TCGCTCCTTAAAGTTGGGGT	rev
**AATFIID** (196 bp)	GTCACAGACTACTATTTGGCGA	fwd
	GATTGTTCATGAGCGCTCGT	rev

[a] 6×His-tag encoding sequences are in bold

[b] Overlapping sequences for Gibson Assembly are underlined

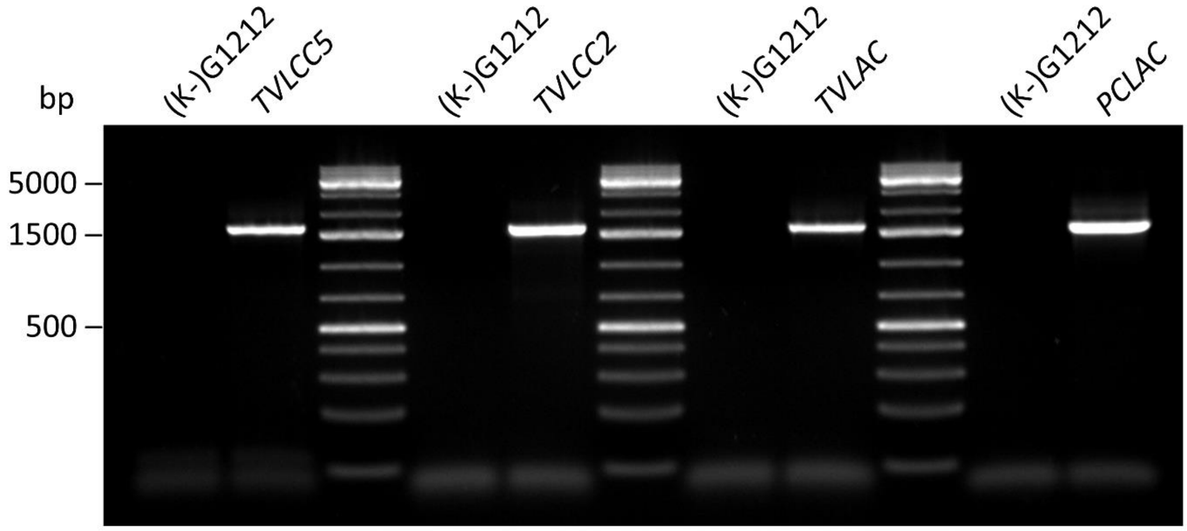

Fig. S1 PCR on genomic DNA of *A. adeninivorans* G1212 strains expressing laccase genes using primers for gene amplification. As negative controls the same PCR reactions were performed using genomic DNA of *A. adeninivorans* G1212(-) as template. Thermo Scientific O'GeneRuler 1 kb Plus served as DNA ladder

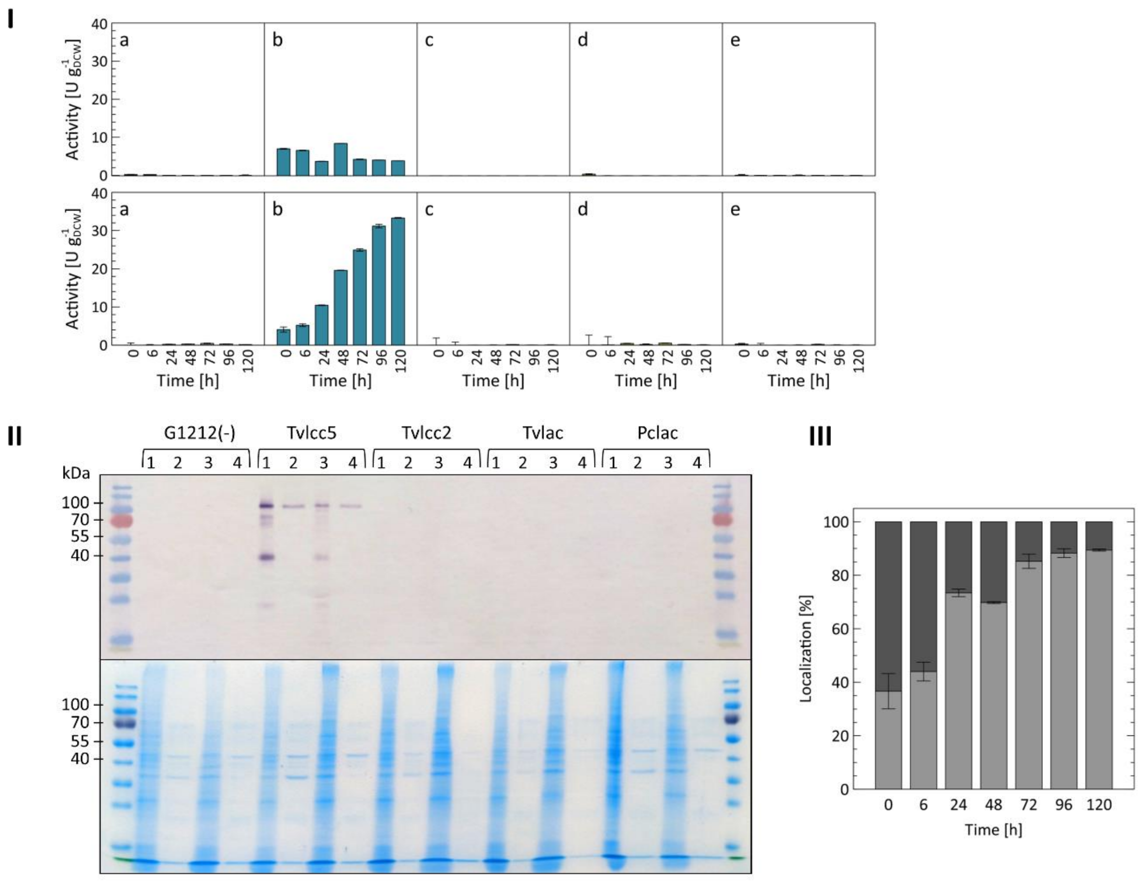

Fig. S2 (**I**) Intracellular (upper row) and extracellular (lower row) laccase activity of (**a**) *A. adeninivorans* G1212 (negative control) and strains expressing (**b**) *TVLCC5*, (**c**)*TVLCC2*, (**d**) *TVLAC*, (**e**) *PCLAC* genes. (**II**) Western-blot and Coomassie-stained SDS-PAGE analysis of intracellular (1, 3) and extracellular (2, 4) fractions isolated after 48 (1, 2) and 120 h (3, 4) of cultivation. (**III**) Distribution of Tvlcc5 protein between intracellular (dark grey) and extracellular (light grey) fraction during 120 h cultivation of *A. adeninivorans* G1212/YRC102-TEF1-TVLCC5-6H

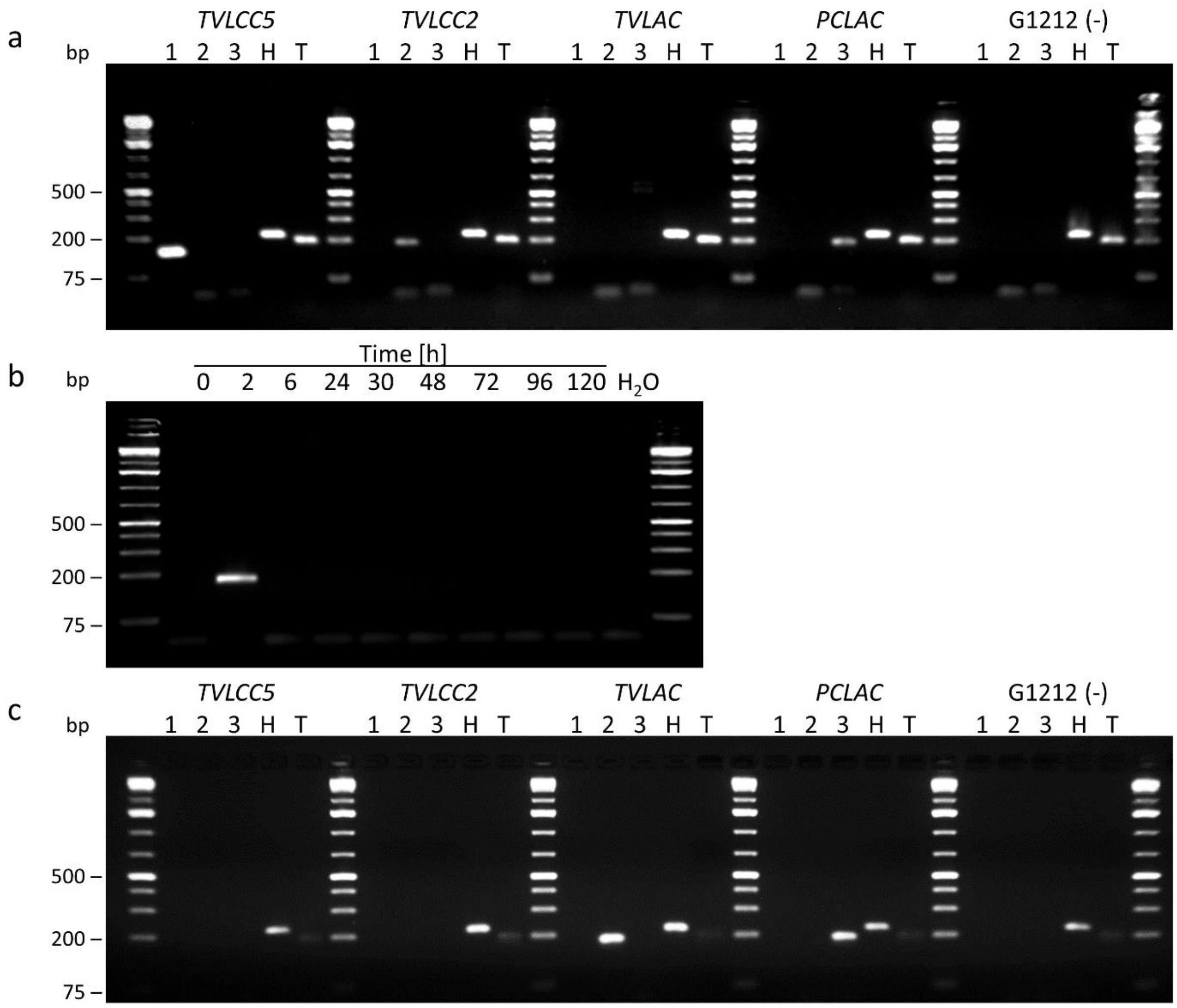

Fig. S3 (**a**) RT-PCR on samples taken from *A. adeninivorans* G1212/YRC102-TVLCC5-6H, *A. adeninivorans* G1212/YRC102-TVLCC2-6H, *A. adeninivorans* G1212/YRC102-TVLAC-6H, *A. adeninivorans* G1212/YRC102-PCLAC-6H, and *A. adeninivorans* G1212/YRC102 after 24 h of cultivation for amplification of fragments of *TVLCC5* (1), *TVLAC* (2), *PCLAC* (3), *AAHEXK* (H), and *AATFIID* (T) using primers shown in Supplemental Table S1. (**b**) RT-PCR on samples taken from *A. adeninivorans* G1212/YRC102-TVLAC-6H after the indicated cultivation time for amplification of a fragment of *TVLAC*. (**c**) RT-PCR on samples taken from the same cultivations after 2 h for amplification of fragments of *TVLCC5* (1), *TVLAC* (2), *PCLAC* (3), *AAHEXK* (H), and *AATFIID* (T) using primers shown in Supplemental Table S1

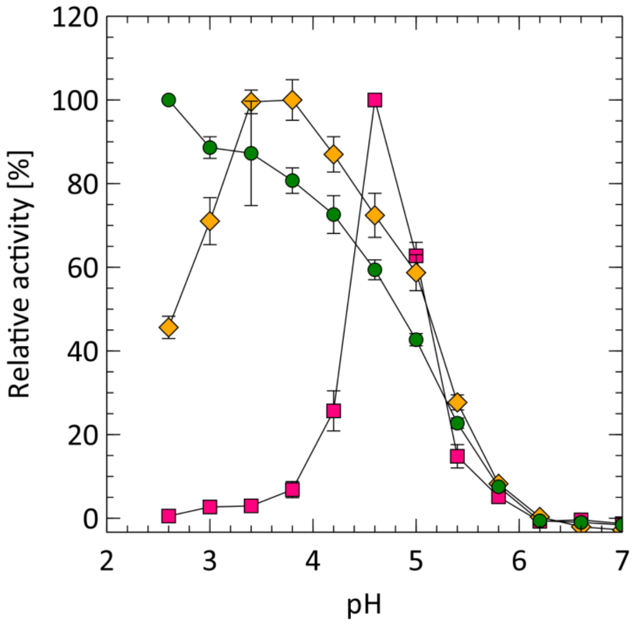

Fig. S4 Effect of pH on the activity of purified Tvlcc5 with ABTS (●), 2,6-DMP (◆), and SGZ (■) as substrate in presence of buffers without equalized ionic strength. Activity was calculated in relation to the maximum value for each substrate

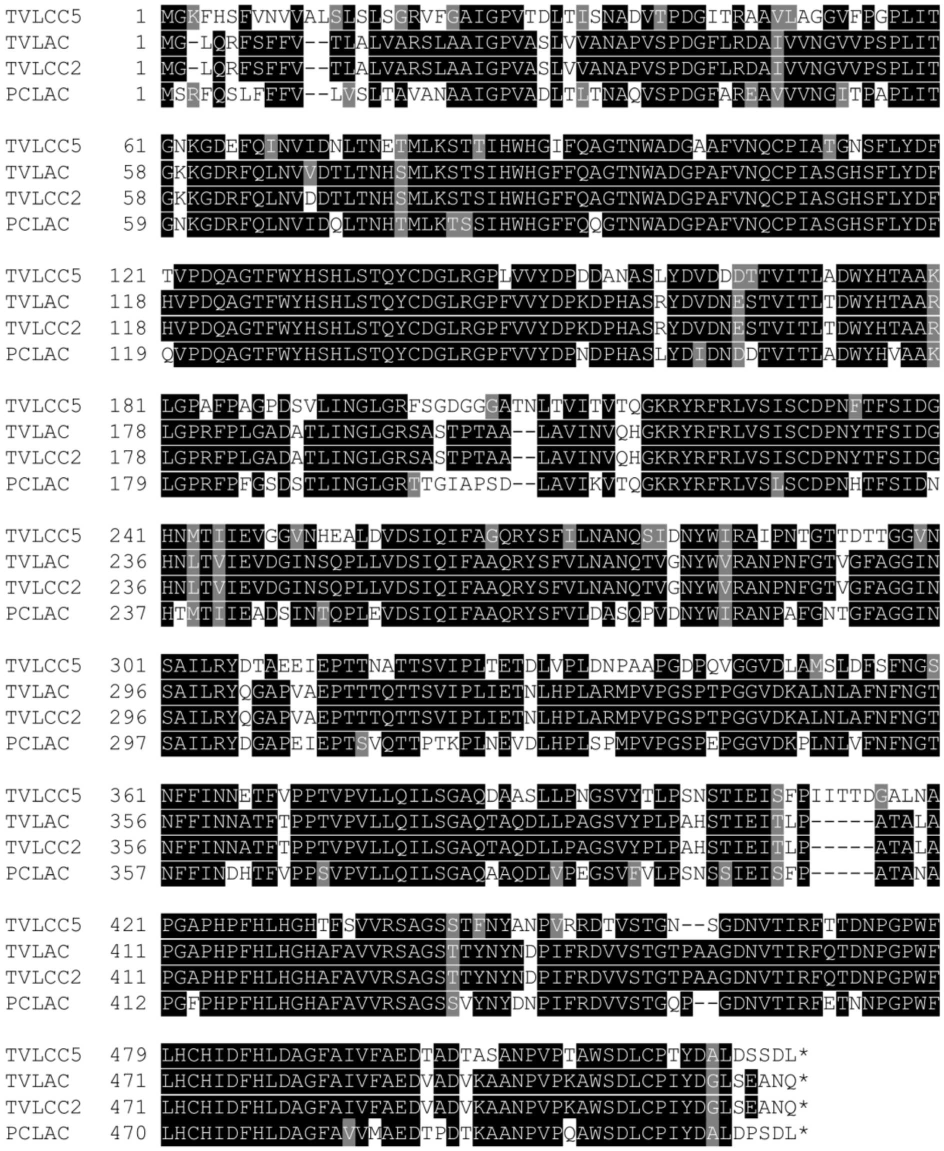

Fig. S5 Alignment of the protein sequences derived from *TVLCC5*, *TVLAC*, *TVLCC2,* and *PCLAC* genes in box shade format. Black box shows identical amino acids. Grey box implies amino acids with similar residues. White text indicates amino acids without similarities
